# Association between olfactory dysfunction and gustatory dysfunction: evidence from the National Health and Nutrition Examination Survey

**DOI:** 10.3389/fpubh.2025.1519290

**Published:** 2025-02-13

**Authors:** Yi Yang, Chao Zhang, Tao Xiong

**Affiliations:** ^1^Department of Pediatrics, West China Second University Hospital, Sichuan University, Chengdu, China; ^2^Key Laboratory of Birth Defects and Related Diseases of Women and Children (Sichuan University) Ministry of Education, Chengdu, China; ^3^West China Biomedical Big Data Center, West China Hospital, Sichuan University, Chengdu, China

**Keywords:** olfaction, gustation, dysfunction, association, measurement, questionnaire, epidemiology, NHANES

## Abstract

**Background:**

Olfactory dysfunction (OD) and gustatory dysfunction (GD) are common among adults, with prevalence increasing significantly in older age groups. Both dysfunctions have negative effects on appetite, nutrition, social functioning and even environmental safety. OD and GD frequently coexist, indicating a possible close association between these conditions. At present, there is a lack of large-sample epidemiological studies on the relationship between OD and GD. Our study aims to investigate the relationship between OD and GD using both measurement and questionnaire data from the 2013–2014 NHANES for US adults aged 40 years and older.

**Methods:**

This cross-sectional study utilized data from the National Health and Nutrition Examination Survey (NHANES). OD and GD were both assessed by measurements and questionnaires. The association between OD and GD was investigated with logistic regression models by adjusting for demographic characteristics, systemic diseases, and diseases of the nose and pharynx. Adjusted odds ratios (aORs) and 95% confidence intervals (CIs) are presented.

**Results:**

Participants with complete olfactory and gustatory measurements and questionnaires (*n* = 2,582) were included. Using whole-mouth measurements, anosmia OD significantly increased the odds of hypogeusia and ageusia GD after adjusting for confounding factors. Similarly, the questionnaire data revealed that OD significantly increased the odds of GD. However, hyposmia OD decreased the odds of hypogeusia GD using the tongue-tip taste measurement.

**Conclusion:**

Our findings highlighted that OD was closely associated with GD in a nationally representative sample of US adults.

## Introduction

Olfaction and gustation are essential senses that significantly contribute to the perception and experience of daily life for human beings (1). Olfactory dysfunction (OD) and gustatory dysfunction (GD) have negative effects on various aspects of an affected population, such as appetite, nutrition, social functioning, and even environmental safety (2). Recent epidemiological studies have indicated a high prevalence of OD and GD, particularly among older adults, underscoring their importance as public health concerns within gerontology. In a nationwide household health survey involving 142.5 million adult Americans, approximately 10.6% of respondents self-reported OD, while 5.3% self-reported GD (3). The prevalence of dysfunction increased with age: OD affected 24.5% of adults aged 53–97, reaching 62.5% in those aged 80–97 (4), while severe GD was reported in 14.8% of adults aged 57–85 (5). With aging, taste and smell decline significantly due to cumulative damage and a reduction in the number of olfactory and gustatory receptor cells, as well as changes in neural responsiveness (6–8). Consequently, research on OD and GD is of great significance and has recently become a hot topic.

The etiology of OD and GD is not fully understood, and multiple risk factors contribute to their development (9). These factors include systemic diseases [such as metabolic and endocrine disorders (10) and neurological disorders (11)], diseases of the nose and pharynx [including inflammation of the nasal meatus and sinuses (12), head trauma and surgery (13)], environmental factors [such as chemical exposures (14)], and even normal aging (15). These factors interact and play important roles in the development of both dysfunctions. With an aging global population and increasing environmental pollution, the incidence of OD and GD is expected to continue to rise (8). Therefore, gaining deeper insights into the relationship between OD and GD will shed new light on our understanding of the human olfactory and gustatory systems.

Preliminary studies supported that there may be a relationship between OD and GD. It has been demonstrated that OD and GD often coexist within one patient (16). Importantly, olfaction and gustation both rely on the same central nervous site, located at the orbitofrontal cortex (17). This commonality of neuroanatomical structures of olfaction and gustation could support the association of OD and GD. Moreover, olfactory information can transmit through two pathways: the nose through the anterior nares (orthonasal olfaction) and the mouth through the oropharynx (retronasal olfaction). The latter pathway may be associated with gustation formation, which is currently a popular research topic (18–23).

At present, there is a lack of population-based epidemiological studies examining the correlation between OD and GD. The National Health and Nutrition Examination Survey (NHANES), conducted by the National Center for Health Statistics (NCHS) and Centers for Disease Control and Prevention (CDC), systematically and continuously collects and analyzes health-related data, including the prevalence of OD and GD (24). Therefore, to provide comprehensive epidemiological evidence, this study aimed to investigate the relationship between OD and GD using both measurement and questionnaire data from the 2013–2014 NHANES for US adults aged 40 years and older.

## Methods

### Ethics statement

Participant survey data were acquired from the publicly accessible NHANES database, sanctioned by the National Center for Health Statistics research ethics review board, and predicated upon the acquisition of informed consent from all participants. The conducted research adhered to the principles outlined in the Declaration of Helsinki and received approval from the NCHS Research Ethics Review Board.

### Study participants

**Figure 1:**
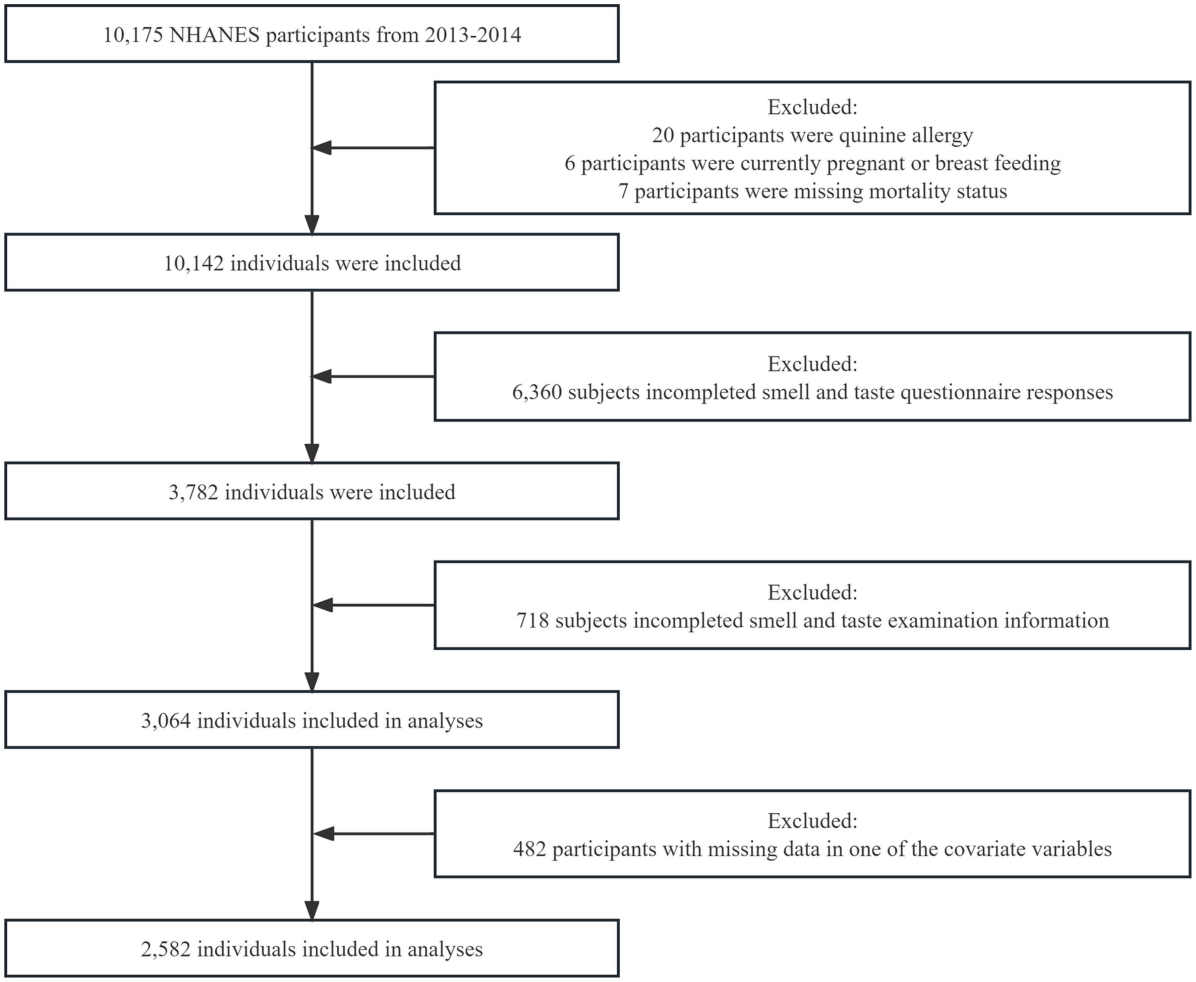
Flow chart of the study population screening.

The NHANES is a national survey program that evaluates the health and nutritional status of both adults and children in the United States. This study analyzed data collected during the 2013–2014 NHANES. The study focused on adults aged 40 years and above who were eligible to participate in the survey. Inclusion Criteria: Participants who completed the 2013–2014 NHANES survey. Exclusion Criteria: (1) Pregnant or breast-feeding participants. (2) Participants with missing mortality status information. (3) Participants allergic to quinine. (4) Participants with incomplete olfactory and gustatory measurement data. (5) Participants with incomplete olfactory and gustatory perception questionnaires. (6) Participants with missing data in one or more covariates. A total of 10,175 participants completed the survey, as reported in previous studies (24, 25). However, 7,111 participants were excluded from the analysis for various reasons, including pregnancy, missing mortality status, allergy to quinine, incomplete olfactory and gustatory measurement information, and incomplete questionnaires on olfactory and gustatory perception. Furthermore, 482 participants with missing data in one or more of the covariate variables were also excluded from the analysis. These exclusions were necessary to ensure the quality and reliability of the data used in this analysis. Consequently, a final sample size of 2,582 participants was included in this analysis (Figure 1). The NHANES protocol was approved by the National Center for Health Statistics (NCHS) institutional review board, and written informed consent was obtained from all participants.

### Olfactory and gustatory measurements

The NHANES olfactory and gustatory measurement protocol consisted of smell testing with the pocket smell test (PST) and taste testing for salt (NaCl) and quinine tastes. The tests were conducted jointly by NHANES health technicians and expert consultants, under the supervision and regular monitoring of the chief health technician, NCHS staff, and the project lead from the data collection contractor (25).

Measured OD (mOD) comprises hyposmia and anosmia. Hyposmia denotes a diminished sense of smell, whereas anosmia refers to a complete loss of smell function (26). The PST is a modified 8-item odor identification test that includes food odors (strawberry, chocolate, onion, and grape), warning odors (natural gas and smoke), and common household odors (leather and soap). The participants were asked to identify the smell out of four given choices presented for each item. Every correct identification is counted as a “point” with a resulting score between 0 and 8. Our study defines mOD as individuals who obtained a score of less than 6 on the PST, indicating incorrect identification of three or more odors. Subsequently, the mOD condition can be further subcategorized into hyposmia (score range of 4–5) and anosmia (score of 3 or below) (27, 28).

Measured GD (mGD) can present in two distinct forms: hypogeusia and ageusia. Hypogeusia refers to a diminished ability to perceive one or more specific tastants, while ageusia denotes a complete absence of gustatory function (29). The gustatory measurements utilized in this study encompassed tongue-tip taste measurement (mGD-t) and whole-mouth taste measurement (mGD-w). Participants were exposed to 1 mM quinine as a bitter taste and subsequently to 1 M NaCl as a salty taste. In the mGD-t procedure, participants applied a tastant attached to a cotton swab to the tip of their tongue in a fixed presentation order. They were then asked to extend their tongue and identify the taste as salty, bitter, a different taste, or no taste. Following this, in the mGD-w protocol, participants were given the opportunity to sip either 1 mM quinine or 1 M NaCl in one of two randomly assigned sequences. Between each test, participants rinsed their mouths with tap water and identified the taste as salty, bitter, a different taste, or no taste. The criteria for determining mGD-t and mGD-w involved the inability to correctly identify quinine or NaCl in either the tongue-tip or whole-mouth tests, respectively (12). Hypogeusia was defined as an erroneous identification on either the quinine or salt test, while ageusia was characterized by incorrect identifications on both quinine and salt tests. We selected only bitter and salty tastes for the following reasons: Firstly, both bitter and salty tastes are representative. For example, bitter taste involves many different receptors (such as the TAS2R family), which can reflect the overall sensitivity of the taste system (30). Salty taste perception is relatively simple and relies on specific ion channels, such as ENaC, which represents the function of ion-based taste (31). Secondly, in existing studies, bitter and salty tastes are widely recognized as key indicators for taste testing (32, 33).

Since NHANES is a large-scale survey conducted on a broad population, using bitter and salty tastes as the means of taste testing ensures acceptability among participants, thereby ensuring the efficiency and consistency of data collection (18). Since the NHANES database includes only bitter and salty taste modalities for testing, we were limited to these two types of tests.

### The olfactory and gustatory questionnaire

The olfactory and gustatory questionnaire section (34) was utilized to gather interview data regarding self-reported olfactory and gustatory abilities, specific symptoms, relevant diseases associated with olfactory and gustatory dysfunctions, and data on potential risk factors for OD and GD.

Olfactory dysfunction by questionnaires (qOD) was defined as one positive answer to any of the following questions: (1) “During the past 12 months, have you had a problem with your ability to smell?” (2) “How would you rate your ability to smell now compared to when you were 25 years old?” (3) “Do you sometimes smell an unpleasant, bad or burning odor when nothing is there?.” GD by questionnaire (qGD) was defined as a positive answer to any of the following questions: (1) “During the past 12 months, have you had a problem with your ability to taste sweet, sour, salty or bitter foods and drinks?” (2) “Is your ability to taste food flavors such as chocolate, vanilla or strawberry as good as when you were 25 years old?” (3) “During the past 12 months, have you had a taste or other sensation in your mouth that does not go away?” (34).

### Demographic and disease-related risk factors

Participant data were collected through NHANES measurements and a questionnaire (Supplementary Table S1) (24). The questionnaire included inquiries such as age, sex, race, education level, and family income-to-poverty ratio (PIR), categorized as low (PIR < 1.3), middle (1.3 ≤ PIR ≤ 3.5), and high (PIR > 3.5) (35). Additional information was gathered on smoking status, alcohol consumption, self-reported chronic diseases (diabetes, congestive heart failure, coronary heart disease, angina pectoris, heart attack, stroke, asthma, cancer or malignancy, and depression), and factors that might influence olfactory and gustatory ability, including frequent nasal congestion, head injury, tonsillectomy, broken nose or serious injury to the face or skull, and sinus infection. Symptoms of depression were assessed using the nine-item Patient Health Questionnaire scale (PHQ-9) with a possible range of 0–27. A cutoff point of ≥10 was applied to identify participants with depression (1).

### Statistical analysis

Continuous variables are presented as the mean ± standard deviation if normally distributed. Otherwise, skewed data are presented as the median (range). Categorical variables are represented as frequencies and percentages. Univariate analysis of variables between groups for continuous and categorical variables was performed using Student’s t tests and Pearson’s chi-square test, respectively.

All statistical analyses were performed using R (Version 4.2.3 for Windows). Considering the complex survey design of NHANES, we used weighted logistics during odds ratio and *p*-value estimation (svyVGM package for multinomial LR, survey package for binomial LR). To avoid sample reduction, we used indicator variables for missing categorical variables and multiple imputation for continuous variables using the mice package.

The association between OD and GD was investigated with logistic regression models. A *p*-value less than 0.05 was considered to indicate statistical significance. We calculated adjusted odds ratios (aORs) and 95% confidence intervals (CIs) after weighting for the sampling distribution of the population. Covariate adjustments were designed for the following four models: the crude (unadjusted) model; Model 1, which was adjusted for demographic characteristics (sex, age, race, education level, income, current smoker, and alcohol drinks); Model 2, which was adjusted for Model 1 + systemic diseases (BMI ≥ 30, high blood pressure, diabetes, asthma, congestive heart failure, coronary heart disease, angina pectoris, heart attack, stroke, cancer or malignancy, and depression); and Model 3, which was adjusted for Model 2 + diseases of the nose and pharynx (cold/flu/dry mouth in the past 12 months, nasal congestion, tonsils removed, head injury/loss of consciousness, broken nose/serious injury to face/skull, and sinus infections).

## Results

In 2013–2014, a total of 2,582 adult NHANES participants completed olfactory and gustatory testing (both measurements and questionnaire). In the mOD group, 2.9% of the participants suffered from anosmia, and 12.0% suffered from hyposmia (Supplementary Table S2). In mGD-t group, 12.4% had ageusia, while 53.0% had hypogeusia (36). In mGD-w group, 0.7% had ageusia, while 18.3% had hypogeusia (Supplementary Table S3). The overall estimated prevalence of qOD was 20.7%, whereas it was 13.4% for qGD (Supplementary Table S4).

The baseline population characteristics of individuals with mGD-w are presented in Table 1. A total of 2,582 participants were included, of whom 473 (18.3%) had hypogeusia of mGD-w and 19 (0.7%) had ageusia of mGD-w (Supplementary Table S3). Among the total population, 48.8% were male, and 47.7% were aged over 60 years. The major ethnicities were non-Hispanic White (48.5%) and non-Hispanic Black (20.4%). 58.5% had some college/equivalent and College graduate or above, and 36.6% had a household income with a PIR > 3.5. Additionally, 61.2% had a BMI less than 30. Current smokers accounted for 17.8%, while 72.9% were drinkers. Hyposmia was present in 12% of the population, and anosmia in 2.9% (Supplementary Table S2). Univariate comparisons show difference in race, education level, BMI, and mOD incidence. The baseline population characteristics of participants with qGD and mGD-t are presented in Supplementary Tables S7, S8, respectively.

**Table 1:** Baseline population characteristics, measured gustatory dysfunction by whole-mouth (mGD-w) (*n* = 2,582).

Characteristics	mGD by whole-mouth (mGD-w)			*P*
	Normal *n* = 2,090	Hypogeusia *n* = 473	Ageusia *n* = 19	
Male gender	1,020(48.8)	226 (47.8)	10 (52.6)	0.868
Age				0.198
40–50	559 (26.7)	153 (32.3)	6 (31.6)	
50–60	533 (25.5)	121 (25.6)	5 (26.3)	
60–70	536 (25.6)	113 (23.9)	3 (15.8)	
>70	462 (22.1)	86 (18.2)	5 (26.3)	
Race				**0.044**
Mexican American	252 (12.1)	56 (11.8)	1 (5.3)	
Non-Hispanic White	1,025 (49.0)	216 (45.7)	10 (52.6)	
Non-Hispanic Black	403 (19.3)	123 (26.0)	3 (15.8)	
Other Race	410 (19.6)	78 (16.5)	5 (26.3)	
Education level				**0.014**
Less than 12th grade with no diploma	392 (18.8)	90 (19.0)	4 (21.1)	
High school graduate/equivalent	451 (21.6)	129 (27.3)	5 (26.3)	
Some college/equivalent	644 (30.8)	142 (30.0)	1 (5.3)	
College graduate or above	603 (28.9)	112 (23.7)	9 (47.4)	
Income				0.114
PIR < 1.3	551 (26.4)	152 (32.1)	5 (26.3)	
1.3 ≤ PIR ≤ 3.5	769 (36.8)	169 (35.7)	8 (42.1)	
PIR > 3.5	770 (36.8)	152 (32.1)	6 (31.6)	
Alcohol drinker	1,522 (72.8)	354 (74.8)	15 (78.9)	0.571
BMI				**0.030**
<30	1,286(61.5)	260(55.0)	12(63.2)	
≥30	804(38.5)	213(45.0)	7(36.8)	
Smoking				0.051
Never	1,111(53.2)	227(48.0)	10 (52.6)	
Former	618(29.6)	143(30.2)	3(15.8)	
Current	361(17.3)	103(21.8)	6(31.6)	
Measured olfactory function				**<0.001**
Normal	1789(85.6)	396(83.7)	12(63.2)	
Hyposmia	245(11.7)	61(12.9)	3(15.8)	
Anosmia	56(2.7)	16(3.4)	4(21.1)	

BMI, body mass index; PIR, Ratio of family income to poverty.

Bold values indicate statistical significance (*P* < 0.05).

In Table 2, for the unadjusted model, anosmia of mOD strongly increased the odds of hypogeusia of mGD-w (OR: 11.29, 95% CI: 4.55–28.01) or ageusia of mGD-w (OR: 10.78, 95% CI: 3.86–30.08). After adjusting for confounding factors in Model 1 (demographic characters), Model 2 (Model 1 + systemic diseases), and Model 3 (Model 2 + diseases of the nose and pharynx), anosmia of mOD significantly increased the odds of hypogeusia of mGD-w (aOR:16.16, 95% CI:4.18–42.29; aOR: 18.18, 95% CI: 5.95–55.53; aOR:17.65, 95% CI:5.49–56.74) or ageusia of mGD-w (aOR:14.09, 95% CI:4.66–42.60; aOR: 15.64, 95% CI: 5.04–48.55; aOR:15.83, 95% CI:5.03–49.89), respectively. There is no association between hyposmia of mOD and mGD-t (Table 3) or between anosmia of mOD and mGD-w (Table 4). Interestingly, for the unadjusted model, we found that hyposmia of mOD decreased the odds of hypogeusia of mGD-t (OR: 0.63, 95% CI: 0.46–0.86), which persisted after model adjustment (aOR: 0.69, 95% CI: 0.50–0.95; aOR: 0.68, 95% CI: 0.50–0.94; aOR: 0.68, 95% CI: 0.49–0.94), as shown in Table 5.

**Table 2:** Association between anosmia of measured olfactory dysfunction (mOD) and gustatory dysfunction by whole-mouth taste measurement (mGD-w).

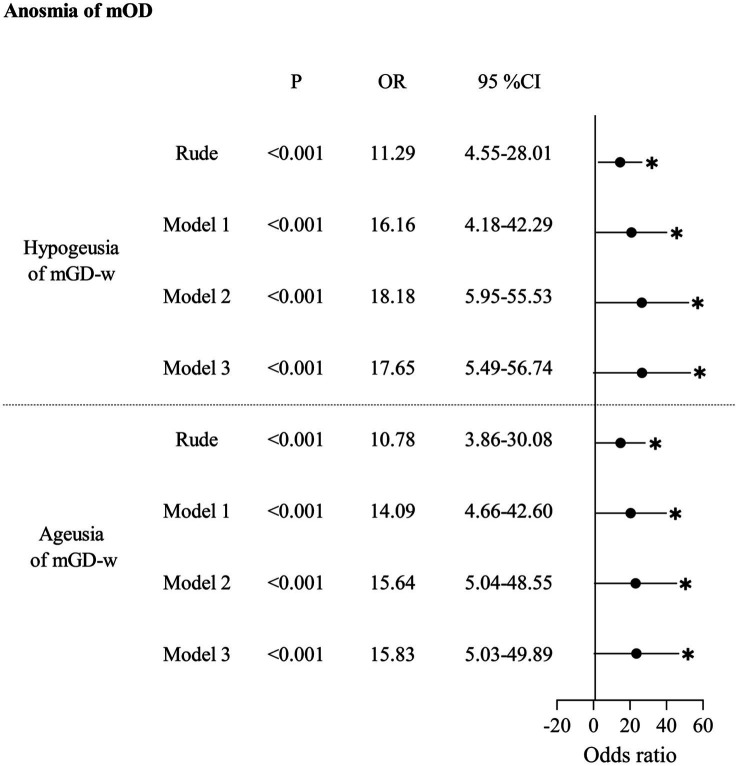

Model 1 was adjusted for: gender, age, race, education level, income, current smoker, and alcohol drinks.

Model 2 was adjusted for: Model 1 + BMI ≥ 30, high blood pressure, diabetes, asthma, congestive heart failure, coronary heart disease, angina pectoris, heart attack, stroke, cancer or malignancy, and depression.

Model 3 was adjusted for: Model 2 + cold/flu/dry mouth in past 12 months, nasal congestion, tonsils removed, head injury/loss of consciousness, broke nose/serious injury to face/skull, and sinus infections.

Data are presented as ORs with 95% confidence intervals. Black error bars correspond to 95% confidence intervals, center for the error bars correspond to points estimate of ORs. Black stars denote *p* < 0.05.

mOD: measured olfactory dysfunction.

mGD-w: whole-mouth taste measurement.

**Table 3:** Association between anosmia of measured olfactory dysfunction (mOD) and gustatory dysfunction by tongue-tip taste measurement (mGD-t).

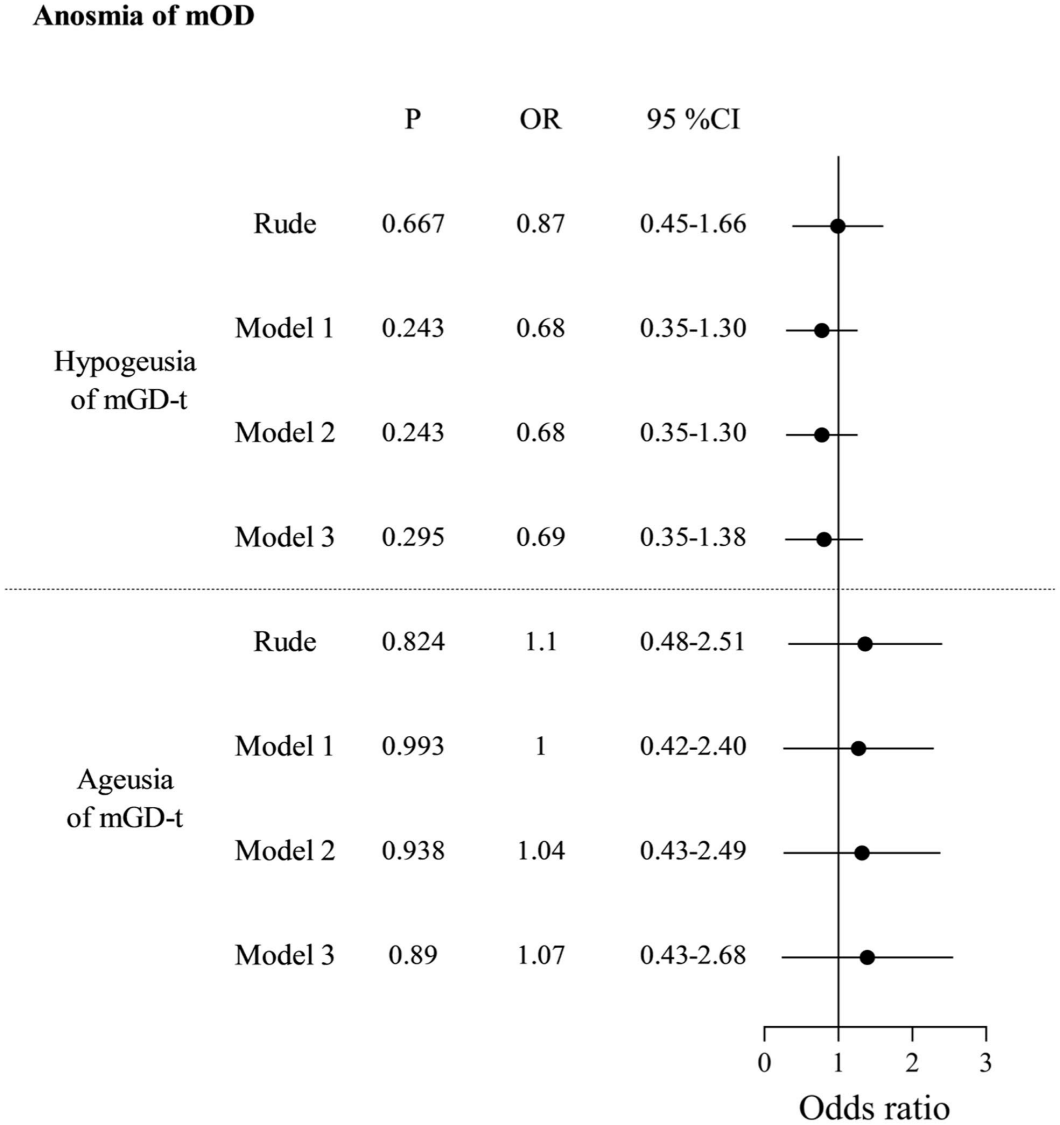

Model 1 was adjusted for: gender, age, race, education level, income, current smoker, and alcohol drinks.

Model 2 was adjusted for: Model 1 + BMI ≥ 30, high blood pressure, diabetes, asthma, congestive heart failure, coronary heart disease, angina pectoris, heart attack, stroke, cancer or malignancy, and depression.

Model 3 was adjusted for: Model 2 + cold/flu/dry mouth in past 12 months, nasal congestion, tonsils removed, head injury/loss of consciousness, broke nose/serious injury to face/skull, and sinus infections.

Data are presented as ORs with 95% confidence intervals. Black error bars correspond to 95% confidence intervals, center for the error bars correspond to points estimate of ORs.

mOD: measured olfactory dysfunction.

mGD-t: tongue-tip taste measurement.

**Table 4:** Association between hyposmia of measured olfactory dysfunction (mOD) and gustatory dysfunction by whole-mouth taste measurement (mGD-w).

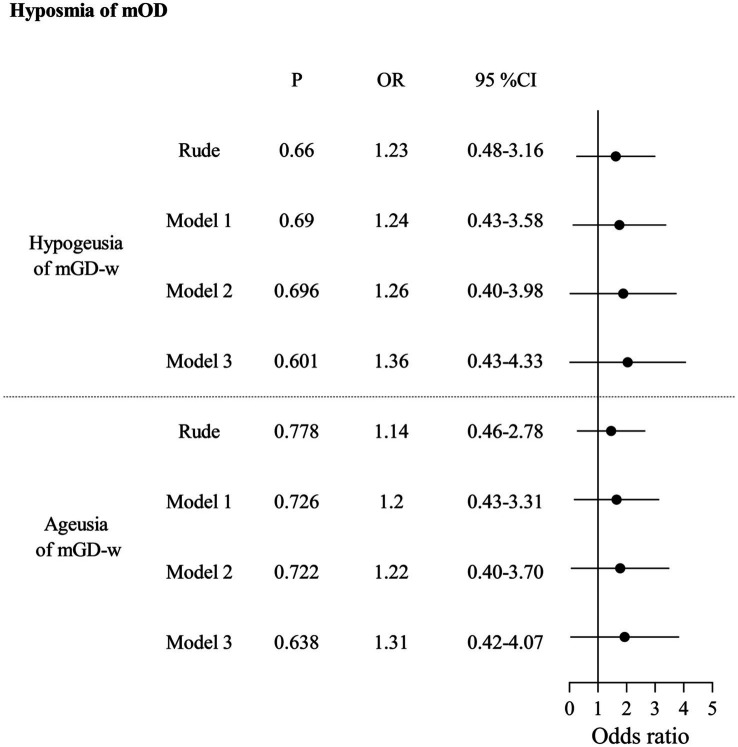

Model 1 was adjusted for: gender, age, race, education level, income, current smoker, and alcohol drinks.

Model 2 was adjusted for: Model 1 + BMI ≥ 30, high blood pressure, diabetes, asthma, congestive heart failure, coronary heart disease, angina pectoris, heart attack, stroke, cancer or malignancy, and depression.

Model 3 was adjusted for: Model 2 + cold/flu/dry mouth in past 12 months, nasal congestion, tonsils removed, head injury/loss of consciousness, broke nose/serious injury to face/skull, and sinus infections.

Data are presented as ORs with 95% confidence intervals. Black error bars correspond to 95% confidence intervals, center for the error bars correspond to points estimate of ORs.

mOD: measured olfactory dysfunction.

mGD-w: whole-mouth taste measurement.

**Table 5:** Association between hyposmia of measured olfactory dysfunction (mOD) and gustatory dysfunction by tongue-tip taste measurement (mGD-t).

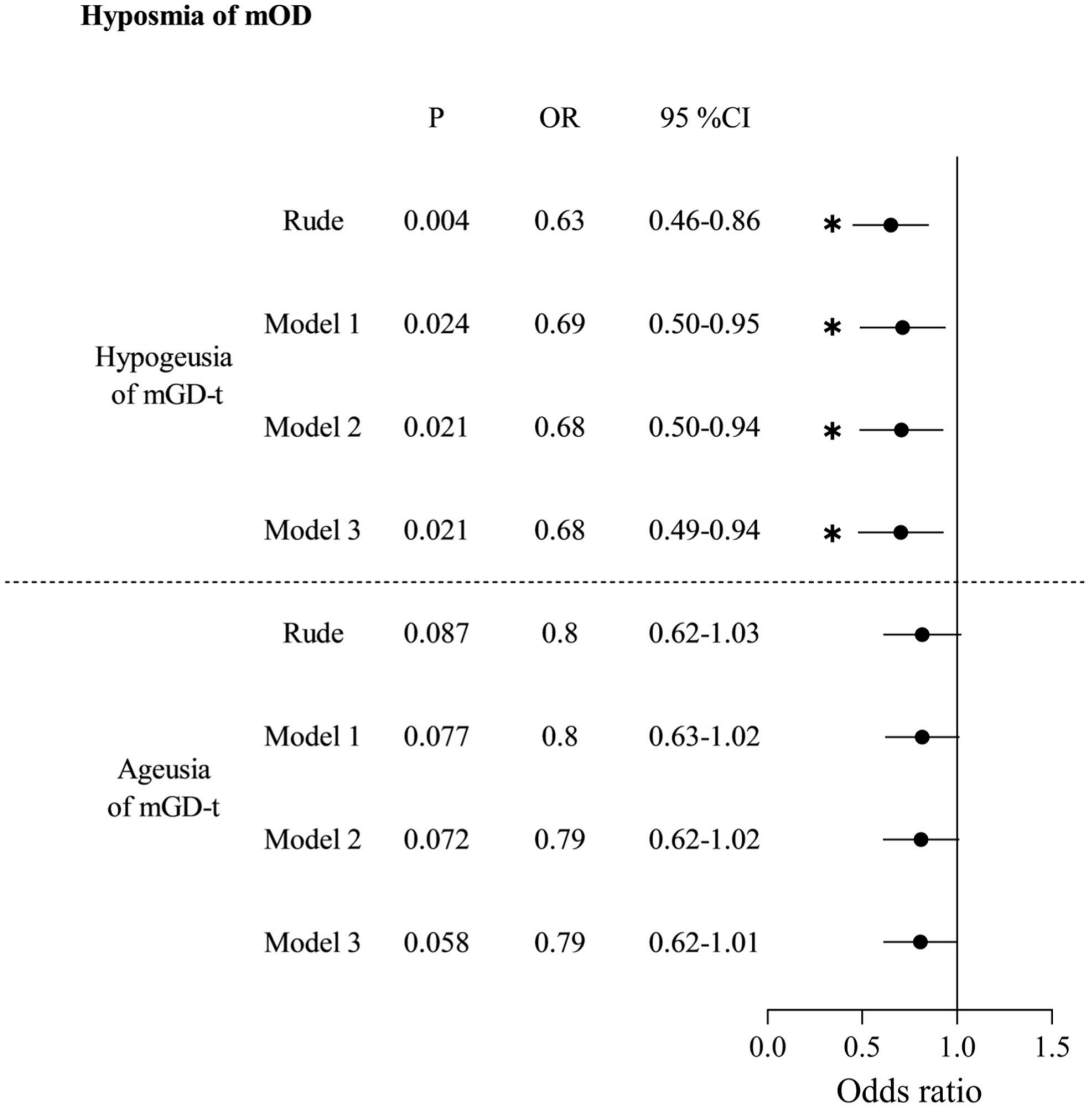

Model 1 was adjusted for: gender, age, race, education level, income, current smoker, and alcohol drinks.

Model 2 was adjusted for: Model 1 + BMI ≥ 30, high blood pressure, diabetes, asthma, congestive heart failure, coronary heart disease, angina pectoris, heart attack, stroke, cancer or malignancy, and depression.

Model 3 was adjusted for: Model 2 + cold/flu/dry mouth in past 12 months, nasal congestion, tonsils removed, head injury/loss of consciousness, broke nose/serious injury to face/skull, and sinus infections.

Data are presented as ORs with 95% confidence intervals. Black error bars correspond to 95% confidence intervals, center for the error bars correspond to points estimate of ORs. Black stars denote *p* < 0.05.

mOD: measured olfactory dysfunction.

mGD-t: tongue-tip taste measurement.

Regarding the association between qOD and qGD (Table 6), qOD significantly increased the odds of qGD, both for the unadjusted model (aOR: 5.90, 95% CI: 4.17–8.35) and for the adjusted model. After adjusting for confounding factors in Model 1, Model 2, and Model 3, qOD significantly increased the odds of qGD (aOR: 5.68, 95% CI: 4.06–7.94; aOR: 5.43, 95% CI: 3.81–7.75; aOR: 5.10, 95% CI: 3.52–7.38, respectively).

**Table 6:** Association between olfactory dysfunction by questionnaire (qOD) and gustatory dysfunction by questionnaire (qGD).

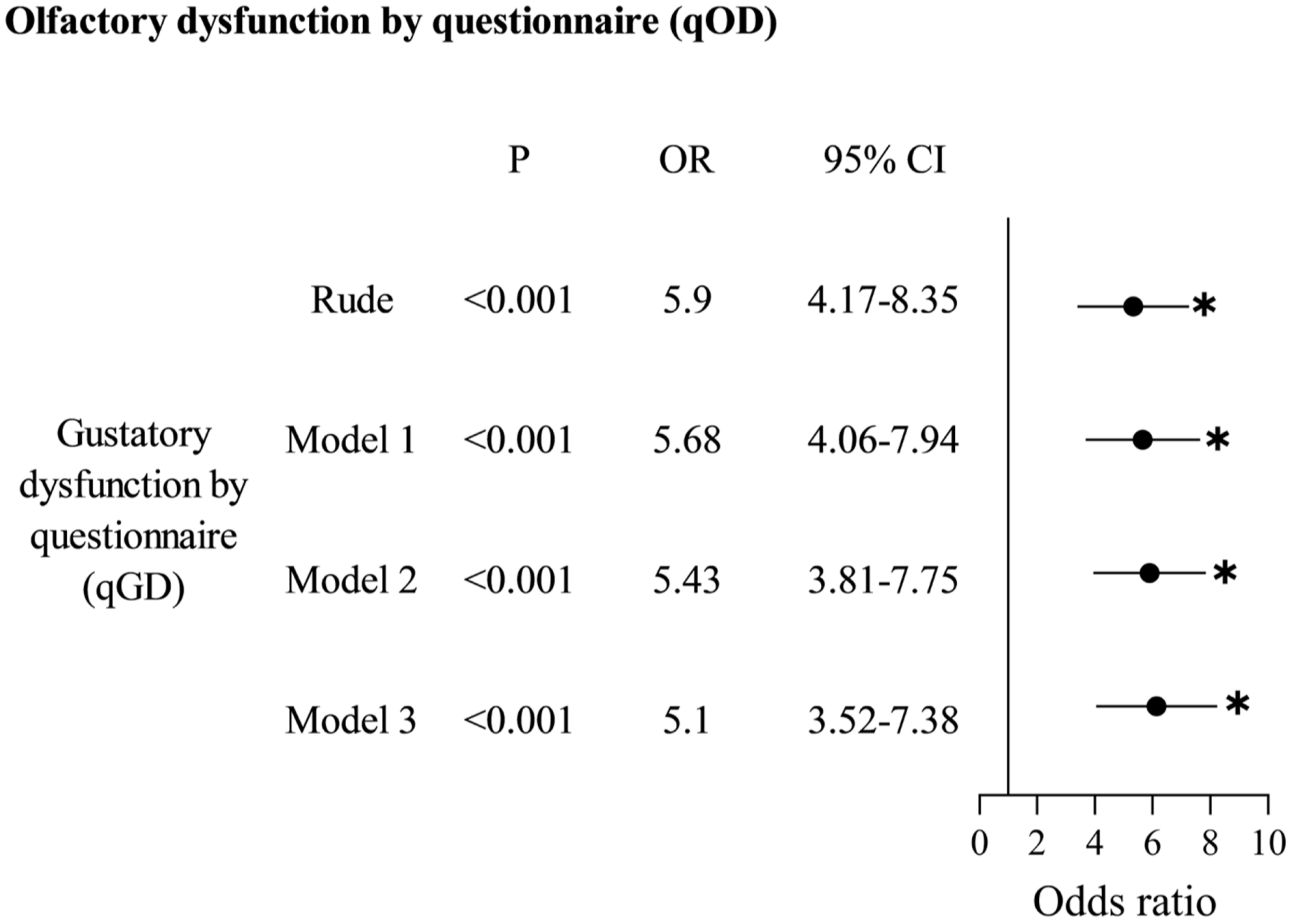

Model 1 was adjusted for: gender, age, race, education level, income, current smoker, and alcohol drinks.

Model 2 was adjusted for: Model 1 + BMI ≥ 30, high blood pressure, diabetes, asthma, congestive heart failure, coronary heart disease, angina pectoris, heart attack, stroke, cancer or malignancy, and depression.

Model 3 was adjusted for: Model 2 + cold/flu/dry mouth in past 12 months, nasal congestion, tonsils removed, head injury/loss of consciousness, broke nose/serious injury to face/skull, and sinus infections.

Data are presented as ORs with 95% confidence intervals. Black error bars correspond to 95% confidence intervals, center for the error bars correspond to points estimate of ORs. Black stars denote *p* < 0.05.

qOD: olfactory dysfunction by questionnaire.

qGD: gustatory dysfunction by questionnaire.

## Discussion

Although several epidemiological studies have reported the prevalence of OD and GD, there is a dearth of research exploring the correlation between these two dysfunctions in a large sample. In this study, we utilized a nationally representative sample of participants from the United States as our target population. For the first time, we provide robust evidence to establish a strong association between OD and GD, which was supported by both objective measurements and questionnaire responses after adjusting for confounding factors. Our measurements revealed a significant association between anosmia of mOD and two mGD-w groups including hypogeusia (aOR: 17.65, 95% CI: 5.49–56.74) and ageusia (aOR: 15.83, 95% CI: 5.03–49.89). Interestingly, hyposmia of mOD decreased the odds of hypogeusia of mGD-t (aOR: 0.68, 95% CI: 0.49–0.94). Additionally, our questionnaire-based analysis demonstrated a significant increase in the odds of experiencing qGD among individuals with qOD (aOR: 5.10, 95% CI: 3.52–7.38).

Due to declining fertility rates and increasing life expectancy (37), the global population is undergoing unprecedented aging. This demographic shift is expected to have profound impacts on various societal aspects, particularly public health (38). Recent data indicate that the proportion of individuals aged 65 years and older accounted for 9.3% of the global population in 2020, and this proportion is projected to rise to 20% by 2050 (39). A hallmark of the aging process is the gradual decline in sensory functions (40). These declines may be interrelated, and investigating their underlying connections to interrupt the “cascade effect” of aging holds significant public health implications. Olfactory and gustatory dysfunctions are prominent issues in the aging population, leading to a substantial decline in quality of life and imposing a considerable burden on public health systems (41). Furthermore, these sensory impairments are closely associated with cognitive decline in older adults (27, 42, 43). From an epidemiological perspective, our study confirms the close association between olfactory and gustatory dysfunctions, providing valuable insights into the mechanisms of aging and potential strategies to delay its progression.

Regarding the diagnosis of OD and GD in this study, we used previously adopted measurements and questionnaires (9, 12, 35). Diagnosis by measurements and questionnaires has the advantage of being reproducible over a 6-month period (44). The measurements for OD and GD diagnosis were reported to be reliable and objective (9, 22, 27, 33, 35), although there is a lack of unified international measurement standards. Questionnaires are easily available, accessible and internationally unified (3, 20).

The close association of OD and GD could be explained by a common integrated central cortex (23). Some studies suggest that gustatory and olfactory information converges at the orbitofrontal cortex on the central nervous system (17). Moreover, some studies have indicated that the processing of retronasal olfactory input occurs in brain regions responsible for taste perception (45).

Previous studies on OD and GD have shown that functional MRI (fMRI) is meaningful in exploring taste and smell phantoms (46), post-traumatic olfactory loss (47), and congenital hyposmia (48). However, its application in the clinical assessment of OD and GD remains limited (49). The primary reason is the significant inter-individual variability in olfactory and gustatory fMRI imaging data, making it unrealistic to consider fMRI as a clinical diagnostic tool for OD and GD (50). Furthermore, studies on the impact of COVID-19 on olfactory and gustatory dysfunctions found that fMRI failed to detect evidence of neuroinvasion (51). Therefore, fMRI for clinical evaluation of OD and GD still needs to overcome these limitations.

Furthermore, the association of OD and GD is also established by the common constitution of olfaction and gustation. Olfactory information is transmitted through two pathways: orthonasal olfaction via the nose (anterior nares) and retronasal olfaction via the mouth (oropharynx) (52). Both pathways convey odors to the olfactory cleft, generating gustatory information. Therefore, when olfaction is impaired, both orthonasal and retronasal olfaction may exhibit similar degrees of impact (53). Previous research has demonstrated that whole mouth gustation is influenced not only by the function of the taste buds, including those on the tip of the tongue (54) and other regions (55), but also by retronasal olfaction (56). Thus, retronasal olfaction plays an important role in whole-mouth gustation.

**Figure 2:**
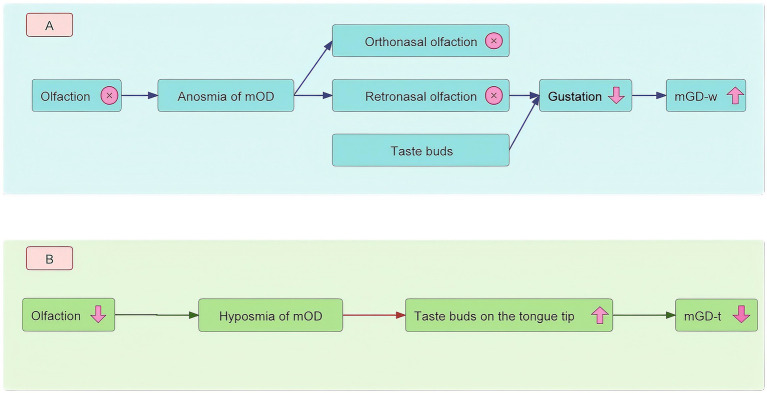
Possible mechanisms for the relationship between olfactory dysfunction and gustatory dysfunction. Panel **(A)** shows that when anosmia of mOD occurs, the complete loss of retronasal olfaction disrupts the generation of gustation, inducing mGD-w. Panel **(B)** shows that hyposmia of mOD induces the function of taste buds on the tongue tip, which represents the decreased odds of mGD-t. mOD, measured olfactory dysfunction; mGD-t, measured tongue-tip gustatory dysfunction; mGD-w, measured whole-mouth gustatory dysfunction.

In this study, we found that anosmia of mODs increased the odds of mGD-w. When anosmia of mODs occurs, the complete loss of retronasal olfaction disrupts the normal generation of gustation, inducing mGD-w (Figure 2A). In our study, after adjusting for confounding factors in Models 1, 2, and 3, anosmia of mOD significantly increased the odds of hypogeusia of mGD-w (aOR: 17.65, 95% CI: 5.49–56.74) and ageusia of mGD-w (aOR: 15.83, 95% CI: 5.03–49.89).

In addition to measurements, the association between OD and GD was supported by questionnaire data. This study also found a strong correlation between self-reported qOD and qGD (aOR: 5.10, 95% CI: 3.52–7.38). This can be explained by the fact that when the OD is severe to the extent of self-perception, it would cause a self-perception of GD to a certain extent.

When hyposmia of mOD occurs, the function of taste buds on the tongue tip increases, which represents the decreased odds of mGD-t (Figure 2B). In our study, we interestingly found that hyposmia of mOD decreased the odds of hypogeusia of mGD-t (aOR: 0.68, 95% CI: 0.49–0.94). The association may be attributed to compensatory mechanisms in sensory systems (57, 58). Some literature suggests that a reduction in olfaction may trigger enhanced responses in the taste buds on the tongue tip (59–61). Therefore, when hyposmia of mOD occurs, the function of taste buds on the tongue tip may increase, which means decreased odds of mGD-t.

Reported OD and GD prevalence are varied. A study of 742 individuals due to preoperative evaluation or subjective OD and/or GD (median age was 55 years) revealed that 65.8% had mOD, 15.9% had mGD, 45.4% had qOD and 6.5% had qGD (22). In a review including a non-clinical population, qOD prevalence estimates ranged from 2.7 to 7.8%, while qGD ranged from 5 to 20% (62). A study including 3,005 adults (older than 55 years) in the US revealed that severe qOD was 2.7% and severe qGD was 14.8% (5). In our study including US adults older than 40 years of age, 7.0% of the population suffered from mOD, 35.0% had mGD-t, 26.7% had mGD-w, 20.1% had qOD, and 13.4% had qGD. The different prevalences may be due to the non-uniform diagnostic criteria for OD and GD (measurements or questionnaires) or the diverse characteristics of the population.

Multiple studies have reported a high comorbidity rate between OD and GD (63). For instance, in a study with a median age of 55 years, among 488 patients with mOD, 93 individuals (19.1%) also exhibited mGD (20). Similarly, in patients attending a specialized smell and taste clinic, 67.8% of the individuals in the qGD population were comorbid with qOD (16). In our study including 385 participants suffering from mOD, the comorbidity rates were 21.8% (84 individuals) for mGD-w and 71.6% (276 individuals) for mGD-t. Additionally, among 535 participants with qOD, 33.5% (179 individuals) had qGD. The consistency of these high comorbidity rates across various studies further supports the close relationship between OD and GD.

This study has several notable strengths. First, it offers a nationally representative estimate of the association between OD and GD based on a substantial sample from the US population. Second, to evaluate that relationship, this study employed both objective measurements and comprehensive questionnaires while carefully controlling for various demographic and disease-related factors. The integration of these two approaches substantially enhances the overall credibility of the research findings. However, certain limitations of this study should be acknowledged. First, due to its cross-sectional nature, the investigation was unable to establish causal relationships between OD and GD. Further longitudinal studies are necessary to explore potential cause-and-effect associations. Second, the gustation assessment in the NHANES only covered bitter and salt stimuli, thereby excluding the evaluation of responses to sweet and sour tastes. In the future, a more comprehensive taste assessment, including salt, bitterness, sweetness, and sourness, is needed to objectively detect GD and measure its degree. On this basis, we explore the relationship between OD and GD.

The influences of OD and GD on disease and health have not been fully elucidated. For future olfactory and gustatory studies, it is imperative to establish internationally uniform diagnostic criteria, incorporating both objective measurements and standardized questionnaires. These studies should encompass diverse populations, including individuals from various age groups and ethnic backgrounds. To establish a causal relationship between OD and GD, long-term follow-up investigations are essential. Such comprehensive research will contribute significantly to our understanding of the impact of both sensory dysfunctions on overall health and disease outcomes.

## Data Availability

The original contributions presented in the study are included in the article/Supplementary material, further inquiries can be directed to the corresponding author.
