# Supplementary material for: Association between olfactory dysfunction and gustatory dysfunction: evidence from the National Health and Nutrition Examination Survey
Source: Front Public Health. 2025 Feb 13;13:1519290. doi: 10.3389/fpubh.2025.1519290 (PMC11864946; doi:10.3389/fpubh.2025.1519290)
Supplement: Supplementary file 1 [file Supplementary_file_1.docx]

**Supplemental table 1 Variables and codes of demographic and related diseases risk factors.**

| **Variable** | | **Data-set** | **Code** |
| --- | --- | --- | --- |
| Respondent number | | All | SEQN |
| Age | | Demographics | RIDAGEYR |
| Gender | | Demographics | RIAGENDR |
| Race | | Demographics | RIDRETH3 |
| Education level | | Demographics | DMDEDUC2 |
| Income | | Demographics | INDFMPIR |
| Alcohol drinker | | Alcohol Use | ALQ101 |
| BMI | | Body Measures | BMXBMI |
| Smoking | | Cigarette Use | SMQ040 |
| Olfactory dysfunction by questionnaire  (qOD) | Had problem with smell past 12 months | Taste & Smell Questionnaire | CSQ010 |
|  | Had change ability to smell since age 25 | Taste & Smell Questionnaire | CSQ020 |
|  | Had phantom odor | Taste & Smell Questionnaire | CSQ040 |
| Gustatory dysfunction by questionnaire  (qGD) | Had problem with taste past 12 months | Taste & Smell Questionnaire | CSQ080 |
|  | Change in ability to taste food flavors | Taste & Smell Questionnaire | CSQ100 |
|  | Persistent taste in mouth past 12 months | Taste & Smell Questionnaire | CSQ110 |
| High blood pressure | Ever told you had high blood pressure | Blood Pressure & Cholesterol | BPQ020 |
| Diabetes | Doctor told you have diabetes | Diabetes | DIQ010 |
| Asthma | Doctor told you have asthma | Medical Conditions | MCQ010 |
| Congestive heart failure | Ever told had congestive heart failure | Medical Conditions | MCQ160b |
| Coronary heart disease | Ever told had coronary heart disease | Medical Conditions | MCQ160c |
| Angina pectoris | Ever told had angina pectoris | Medical Conditions | MCQ160d |
| Heart attack | Ever told had heart attack | Medical Conditions | MCQ160e |
| Stroke | Ever told had stroke | Medical Conditions | MCQ160f |
| Cancer or malignancy | Ever told you had cancer or malignancy | Medical Conditions | MCQ220 |
| Cold/flu | Persistent cold/flu last 12 months | Taste & Smell Questionnaire | CSQ200 |
| Nasal congestion | Frequent nasal congestion in past 12 months | Taste & Smell Questionnaire | CSQ204 |
| Dry mouth | Dry mouth in past 12 months | Taste & Smell Questionnaire | CSQ202 |
| Head injury/loss of consciousness | Ever had head injury/loss of consciousness | Taste & Smell Questionnaire | CSQ240 |
| Tonsils removed | Ever had tonsils removed | Taste & Smell Questionnaire | CSQ220 |
| Broke nose/serious injury to face /skull | Ever had broke nose/serious injury to face/ skull | Taste & Smell Questionnaire | CSQ250 |
| Sinus infections | Ever had two or more sinus infections | Taste & Smell Questionnaire | CSQ260 |
| Quinine allergy | Ever had quinine allergy | Taste & Smell Questionnaire | CSQ245 |
| Pregnant or breast feeding | Currently pregnant or breast feeding | Taste & Smell Questionnaire | CSQ241 |

**Supplemental table 2 The proportion and count of the score of pocket smell test (n = 2,582).**

| **Classification** | **Smell score** | **Count** | **Proportion** | **Count** | **Proportion** |
| --- | --- | --- | --- | --- | --- |
| Anosmia | 0 | 4 | 0.2% | 76 | 2.9% |
|  | 1 | 7 | 0.3% |  |  |
|  | 2 | 26 | 1.0% |  |  |
|  | 3 | 39 | 1.5% |  |  |
| Hyposmia | 4 | 101 | 3.9% | 309 | 12.0% |
|  | 5 | 208 | 8.1% |  |  |
| Normal | 6 | 501 | 19.4% | 2197 | 85.1% |
|  | 7 | 808 | 31.3% |  |  |
|  | 8 | 888 | 34.4% |  |  |

**Supplemental table 3 The proportion and count of tongue-tip taste measurement (mGD-t) and whole-mouth taste measurement (mGD-w) (n = 2,582).**

| **Classification** | | **Count** | **Proportion** |
| --- | --- | --- | --- |
| mGD-t | Ageusia | 320 | 12.4% |
|  | Hypogeusia | 1368 | 53.0% |
|  | Normal | 894 | 34.6% |
| mGD-w | Ageusia | 19 | 0.7% |
|  | Hypogeusia | 472 | 18.3% |
|  | Normal | 2092 | 81.0% |

mGD-t,tongue-tip taste measurement; mGD-w, whole-mouth taste measurement.

**Supplemental table 4 The proportion and count of gustatory dysfunction by questionnaire (qGD) and olfactory dysfunction by questionnaire(qOD) (n = 2,582).**

|  | **Classification** | **Count** | **Proportion** |
| --- | --- | --- | --- |
| qGD | Yes | 345 | 13.4% |
|  | No | 2237 | 86.6% |
| qOD | Yes | 535 | 20.7% |
|  | No | 2047 | 79.3% |

qGD:Gustatory dysfunction by questionnaire; qOD:Olfactory dysfunction by questionnaire.

**Supplemental table 5 The proportion and count of the gustatory dysfunction and olfactory dysfunction by questionnaire (n = 2,582).**

| **Questions** | **Answer** | **Count** | **Proportion** |
| --- | --- | --- | --- |
| During the past 12 months, have you had a problem with your ability to smell? | Yes | 207 | 8.0% |
|  | No | 2375 | 92.0% |
| How would you rate your ability to smell now compared to when you were 25 years old? | Better Now | 170 | 6.6% |
|  | Worse Now | 374 | 14.5% |
|  | No Change | 2038 | 78.9% |
| Do you sometimes smell an unpleasant, bad or burning odor when nothing is there? | Yes | 192 | 7.4% |
|  | No | 2390 | 92.6% |
| During the past 12 months, have you had a problem with your ability to taste sweet, sour, salty or bitter foods and drinks? | Yes | 125 | 4.8% |
|  | No | 2457 | 95.2% |
| Is your ability to taste food flavors such as chocolate, vanilla or strawberry as good as when you were 25 years old? | Yes | 2412 | 93.4% |
|  | No | 170 | 6.6% |
| During the past 12 months, have you had a taste or other sensation in your mouth that does not go away? | Yes | 159 | 6.2% |
|  | No | 2423 | 93.8% |

**Supplemental table 6 The proportion and count of** **taste testing for salt (NaCl) and quinine tastes (n = 2,582).**

| **Taste testing** | **Tongue Tip** | | | | **Whole Mouth** | | | |
| --- | --- | --- | --- | --- | --- | --- | --- | --- |
|  | **1mM Quinine** | | **1M NaCl** | | **1 mM Quinine** | | **1 M NaCl** | |
|  | **Count** | **Proportion** | **Count** | **Proportion** | **Count** | **Proportion** | **Count** | **Proportion** |
| Salty | 83 | 3.2% | 2146 | 83.1% | 27 | 1.0% | 2517 | 97.5% |
| Bitter | 1007 | 39.0% | 96 | 3.7% | 2136 | 82.7% | 25 | 1.0% |
| Something else | 403 | 15.6% | 78 | 3.0% | 282 | 10.9% | 17 | 0.7% |
| No taste | 953 | 36.9% | 80 | 3.1% | 14 | 0.5% | 1 | <0.1% |
| Sour | 136 | 5.3% | 182 | 7.0% | 123 | 4.8% | 22 | 0.9% |

**Supplemental table 7 Baseline Population Characteristics of Gustatory Dysfunction by questionnaire(qGD) (n = 2,582).**

| **Characteristics** | **Gustatory Dysfunction by questionnaire (qGD)** | | **P** |
| --- | --- | --- | --- |
|  | Yes  n =345 | No  n =2,237 |  |
| Male gender | 142(41.2) | 1114(49.8) | **0.003** |
| Age |  |  | 0.086 |
| 40-50 | 89(25.8) | 629(28.1) |  |
| 50-60 | 105(30.4) | 554(24.8) |  |
| 60-70 | 89(25.8) | 563(25.2) |  |
| ＞70 | 62(18.0) | 491(21.9) |  |
| Race |  |  | **0.012** |
| Mexican American | 59(17.1) | 250(11.2) |  |
| Non-Hispanic White | 162(47.0) | 1089(48.7) |  |
| Non-Hispanic Black | 60(17.4) | 469(21.0) |  |
| Other Race | 64(18.6) | 429(19.2) |  |
| Education level |  |  | **<0.001** |
| Less than 12th grade with no diploma | 91(26.4) | 395(17.7) |  |
| High school graduate/equivalent | 72(20.9) | 513(22.9) |  |
| Some college/equivalent | 114(33.0) | 673(30.1) |  |
| College graduate or above | 68(19.7) | 656(29.3) |  |
| Income |  |  | **<0.001** |
| PIR<1.3 | 134(38.8) | 574(25.7) |  |
| 1.3≤PIR≤3.5 | 107(31.0) | 839(37.5) |  |
| PIR>3.5 | 104(30.1) | 824(36.8) |  |
| Alcohol drinker | 245(71.0) | 1,646(73.6) | 0.316 |
| BMI |  |  | **0.073** |
| <30 | 193(55.9) | 1,365(61.0) |  |
| ≥30 | 152(44.1) | 872(39.0) |  |
| Smoking |  |  | 0.159 |
| Never | 166(48.1) | 1,182(52.8) |  |
| Former | 105(30.4) | 659(29.5) |  |
| Current | 74(21.4) | 396(17.7) |  |
| qOD | 179 (51.9) | 356 (15.9) | **<0.001** |

BMI, body mass index; PIR, Ratio of family income to poverty; qOD, Olfactory dysfunction by questionnaire.

**Supplemental table 8 Baseline population characteristics,measured gustatory dysfunction by tongue-tip(mGD-t) (n =2,582).**

| **Characteristics** | **Measured gustatory dysfunction by tongue-tip (mGD-t)** | | | **P** |
| --- | --- | --- | --- | --- |
|  | Normal  n =892 | Hypogeusia  n =1,369 | Ageusia  n =321 |  |
| Male gender | 385(43.2) | 685(50.0) | 186(57.9) | **<0.001** |
| Age |  |  |  | **<0.001** |
| 40-50 | 295(33.1) | 348(25.4) | 75(23.4) |  |
| 50-60 | 222(24.9) | 352(25.7) | 85(26.5) |  |
| 60-70 | 219(24.6) | 348(25.4) | 85(26.5) |  |
| ＞70 | 156(17.5) | 321(23.4) | 76(23.7) |  |
| Race |  |  |  | 0.224 |
| Mexican American | 94(10.5) | 177(12.9) | 38(11.8) |  |
| Non-Hispanic White | 431(48.3) | 655(47.8) | 165(51.4) |  |
| Non-Hispanic Black | 177(19.8) | 284(20.7) | 68(21.2) |  |
| Other Race | 190(21.3) | 253(18.5) | 50(15.6) |  |
| Education level |  |  |  | 0.056 |
| Less than 12th grade with no diploma | 151(16.9) | 273(19.9) | 62(19.3) |  |
| High school graduate/equivalent | 191(21.4) | 311(22.7) | 83(25.9) |  |
| Some college/equivalent | 266(29.8) | 424(31.0) | 97(30.2) |  |
| College graduate or above | 284(31.8) | 361(26.4) | 79(24.6) |  |
| Income |  |  |  | 0.081 |
| PIR<1.3 | 229(25.7) | 374(27.3) | 105(32.7) |  |
| 1.3≤PIR≤3.5 | 324(36.3) | 503(36.7) | 119(37.1) |  |
| PIR>3.5 | 339(38.0) | 492(35.9) | 97(30.2) |  |
| Alcohol drinker | 637(71.4) | 1004(73.3) | 250(77.9) | 0.080 |
| BMI |  |  |  | 0.071 |
| <30 | 545(61.1) | 803(58.7) | 210(65.4) |  |
| ≥30 | 347(38.9) | 566(41.3) | 111(34.6) |  |
| Smoking |  |  |  | **<0.001** |
| Never | 506(56.7) | 710(51.9) | 132(41.1) |  |
| Former | 232(26.0) | 421(30.8) | 111(34.6) |  |
| Current | 154(17.3) | 238(17.4) | 78(24.3) |  |
| Measured olfactory function |  |  |  | **0.014** |
| Normal | 783 (87.8) | 1,155 (84.4) | 259 (80.7) |  |
| Hyposmia | 83 (9.3) | 177 (12.9) | 49 (15.3) |  |
| Anosmia | 26 (2.9) | 37 (2.7) | 13 (4.0) |  |

BMI, body mass index; PIR, Ratio of family income to poverty.
